# Facebook as a Medium for the Support and Enhancement of Ambulatory Care for People With Diabetes: Qualitative Realist Evaluation of a Real-World Trial

**DOI:** 10.2196/18146

**Published:** 2020-09-14

**Authors:** Bryan Cleal, Ingrid Willaing, Mette T Hoybye, Henrik H Thomsen

**Affiliations:** 1 Diabetes Management Research Steno Diabetes Center Copenhagen Copenhagen Denmark; 2 Interacting Minds Center Insitute for Clinical Medicine University of Aarhus Aarhus Denmark; 3 Department of Endocrinology Regional Hospital Viborg Region Midtjylland Viborg Denmark

**Keywords:** online patient-provider interaction, social media, Facebook, realistic evaluation

## Abstract

**Background:**

There is a growing focus on the potential uses, benefits, and limitations of social media in the context of health care communication. In this study, we have sought to evaluate an initiative pioneered at a hospital in Denmark that uses Facebook to support and enhance patient-provider communication about diabetes.

**Objective:**

This paper aims to evaluate the success of the trial according to its initial objectives and to assess its potential scalability.

**Methods:**

The study was undertaken in a clinic for diabetes and hormonal diseases at a large regional hospital in Denmark. Using a realist evaluation approach, we identified 4 key components in the program theory of the initiative, which we formulated as context-mechanism-outcome configurations (eg, complex and iterative chains of causality). These configurations informed data gathering and analysis. Primary data sources were the activity and content in the Facebook group, in the form of posts, likes, and comments, and interviews with patients (n=26) and staff (n=6) at the clinic.

**Results:**

New developments in diabetes technology were the most popular posts in the forum, judged by number of likes and comments. Otherwise, information specific to the clinic received the most attention. All 4 components of the program theory were compromised to varying degrees, either as a result of failings in the anticipated mechanisms of change or contextual factors derived from the mode of implementation.

**Conclusions:**

Social media serves well as a conduit for imagining positive change, but this can be a strength and weakness when attempting to enact change via concrete interventions, where stakeholder expectations may be unreasonably high or incompatible. Nonetheless, such initiatives may possess intangible benefits difficult to measure in terms of cost-effectiveness.

## Introduction

### Background

Diabetes mellitus is a complex and multifarious health condition that impacts millions of people globally, giving rise to both personal and societal costs on a large scale [1]. In recent years, incidence and prevalence of diabetes mellitus has been on the increase, with more people than ever before confronting the day-to-day challenges associated with diabetes management [2]. This increase puts pressure on individuals, but also challenges health care systems. More and more resources within health care are consumed by the treatment of diabetes mellitus and its complications [3]. In this climate, innovation, both technical and organizational, is widely seen as key to confronting challenges anticipated in the future.

Social media platforms are oft-touted as one possible area of innovation that can be of benefit within health care [4-6]. Use of social media has, for example, been shown to enhance relationships with health care professionals (HCPs), with people feeling empowered and better able to engage in shared decision making about their care [7,8]. In the case of the social media platform Facebook, it has been shown that online exchanges between patients and relatives can influence treatment decisions and emotional support in everyday life [9], though some of the factual content of the information being exchanged was deemed to be questionable from a strictly clinical perspective [10].

The recent emergence and growth of the diabetes online community (DOC) presents opportunities and challenges to health care professionals and health care systems [11-16]. People with diabetes can now interact with one another irrespective of time or place, and this impacts how knowledge about diabetes is acquired and exchanged [17]. For people with diabetes who are willing and able to participate in the DOC, there is apparently much to be gained by this development. The rapid pace of change observed with respect to the communication between people living with chronic conditions such as type 1 and type 2 diabetes is not yet fully matched by concomitant changes in modes of communication between health care professionals and the people they provide care for.

Traditional roles in health care communication are thrown into flux by the advent of social media [18], and HCPs and health care systems are still struggling to define or redefine their position. The spread of social media creates new ethical dilemmas within health care [19]. Taking the specific case of Facebook, a significant concern among HCPs is the potential threat it poses to personal privacy and a fear that the private sphere will be overwhelmed by the professional sphere [20]. In addition, there is a concern that social media forums foster inaccurate information, posing both practical and ethical dilemmas to HCPs interested in using these media as channels for communication.

There is a growing focus on the potential uses, benefits, and limitations of social media in the context of health care communication [4]. In the case of type 1 diabetes, it has been proposed that, where appropriate, clinicians need to be more proactive in supporting their patients to engage with social media [21] and that exchanges on social media can provide a potential source of information for the health care professions, which can be used to inform new health-related interventions [22]. Where Facebook has been used to engage patients, it has generally not been used to interact directly with them but more commonly to provide general guidance and correct what HCPs perceive to be misleading or spurious online information, as described in Benetoli et al [23].

Aside from the ethical and legal concerns associated with social media–facilitated health care communication [24,25], a further limitation for promoting such engagement by health care systems and HCPs is the fact that, with some exceptions [26,27], the use of Facebook by HCPs has not been associated with outcomes justifying the use of time and resources required to sustain this type of intervention [28]. This is striking because, at face value, Facebook is a medium that is well and widely established in countries like Denmark, where it is estimated that there are up to 3 million regular users in a country of approximately 5 million inhabitants. Part of the challenge here rests in the fact that social media interventions are essentially complex, since the component parts are difficult to isolate from one another and from other wider contexts, thereby challenging traditional research and evaluation methods [29].

In this study we have sought to evaluate an initiative, pioneered at a hospital in Denmark, to use Facebook to communicate directly from HCP to people with diabetes. At the time our evaluation was undertaken, the Facebook group being used to facilitate this initiative had been active for approximately 18 months. At the outset, the initiative was not designed as an intervention, the impact of which might be directly or indirectly measured. Nonetheless, after seeing membership of the Facebook group grow substantially from its inception and in view of the effort required to maintain the group, the owner of the initiative (the head physician) considered that it was timely to determine whether the group was achieving the objectives for which it was developed. In view of the difficulties noted above concerning evaluation of such initiatives, it was agreed by the partners involved in this work that the optimal approach would be to undertake a theory-driven evaluation. Theory-driven evaluation represents an ideal approach to the appraisal of complex real-world interventions [30]. Evaluation thus proceeds from an identification of the theories that have informed the development and implementation of the intervention, and these theories are subsequently used to shape the approach of the evaluation, determining the primary points of focus and the questions that need to be posed.

In this study, we chose to apply a particular form of theory-driven evaluation known as realist evaluation (RE) [31]. This choice was influenced by the fact that this approach is well suited for social interventions, where outcomes are determined by stakeholder actions and interactions [32], a point very apposite to the topic we were focusing upon. Likewise, RE is particularly concerned with both the psychological and motivational impact of initiatives that lead to change [33], and this focus is not only important in itself for the purposes of our specific evaluation but also more broadly in terms of the lessons that the evaluation we present in this study might have for other similar initiatives.

### Goal of This Study

This study aims to evaluate the success of a concrete intervention using Facebook as a means to support and enhance ambulatory care among people with type 1 and type 2 diabetes. Additionally, we sought to identify more generic factors influencing the use and uptake of social media in the context of health care. Finally, we sought to apply and exemplify the use of realist evaluation as a methodology for apprehending complex outcomes within a complex, real-world intervention.

## Methods

### The Setting

The study was undertaken in a clinic for diabetes and hormonal diseases, which is part of a large regional hospital in provincial Denmark. The outpatient clinic caters to people with both type 1 and type 2 diabetes, with a capacity for approximately 2500 consultations per annum for people with diabetes. The clinic employs 3 chief consultants, 2 residents, 5 diabetes nurses, and 5 dieticians.

### The Virtual Setting

The Facebook group, Diabetes Viborg (DIAVIB), was established by a consultant endocrinologist in the clinic in January 2017. DIAVIB was not established with an explicit set of aims and objectives, but the initiative was motivated by the interests and concerns of this consultant endocrinologist regarding the use of social media by people with diabetes. It was set up as a closed group, requiring registration by potential members, and it targeted people with diabetes, their family members, and anyone with an interest in diabetes. The Facebook group focused primarily on users of the clinic but also stated that it was open to anyone with an interest in diabetes. At the time of our evaluation, there were approximately 500 registered members, of whom approximately two-thirds were women (at the time of writing, this figure is now 630, with the sex distribution unchanged). In terms of age distribution, the lowest proportion of users was seen in the age range of 18 to 24 years, with the next lowest in the 65+ years age range. The majority of users lived in the catchment area of the clinic. Communication on DIAVIB was almost exclusively conducted in Danish, although some links were provided to external content that was only available in English.

### Data Material and Participants

The study draws upon 2 primary data sources: the activity and content on DIAVIB, in the form of posts, likes, and comments observed over the period from June 1, 2017, to August 22, 2018, and interviews with patients and staff at the clinic. Interview participants were sought via posts on DIAVIB, a leaflet posted in the clinic, and by a nurse in the clinic, who phoned people visiting the clinic on the days on which interviews were planned. We sought to recruit a representative sample of the clinic’s overall population, seeking variation according to age, gender, social class, and both users and nonusers of DIAVIB. Patient characteristics can be seen in Table 1.

Two potential participants declined the invitation to participate when asked directly, primarily due to a general dislike of social media and practical issues with available interview times. In addition to the consultant who founded DIAVIB, other HCPs in the clinic were also interviewed, namely 2 nurses, 2 consultants, and 2 dieticians.

**Table 1:** Study participant characteristics (N=26).

Characteristic			Value
Age (years), median (range)			48.1 (19-77)
Diabetes duration (years), median (range)			13 (3-57)
**Diabetes type, n (%)**			
	Type 1	15 (58)	
	Type 2	11 (42)	
**Gender, n (%)**			
	Male	12 (44)	
	Female	15 (56)	
BMI (kg/m^2^), median (range)			28.7 (21-42)
**HbA _1c _^a^ (mmol/mol), median (range)**			
	Type 1	67.6 (46-89)	
	Type 2	57.2 (40-71)	
Existing DIAVIB^b^ member (yes), n (%)			11 (42)
**Employment status, n (%)**			
	In employment	15 (58)	
	Unemployed	1 (4)	
	Pensioned	3 (12)	
	Disability pensioned	5 (19)	
	Student	2 (8)	

^a^HbA_1c_: glycosylated hemoglobin (used to measure average blood glucose levels over time).

^b^DIAVIB: Diabetes Viborg.

### Data Analysis

In cases where there is no clear set of theoretical principles explicitly coupled to an intervention, the first task for evaluators working with theory-driven approaches is to articulate a program theory. This is undertaken in collaboration with those who have developed the intervention, in this case the consultant at the clinic. With numerous informal discussions and a 2-hour semistructured interview, we initially identified 4 distinct objectives, which we were then able to investigate and assess.

In addition to the interview data, we also gathered and analyzed data from DIAVIB itself. These data were analyzed in terms of their general characteristics (eg, a comment, a question, a like, etc) and in terms of their content. BC undertook the first analysis and thematization of the content, and this was subsequently discussed and consensually verified within the author group. It was relatively straightforward to achieve high levels of consensus within the author group because the content being analyzed was, for the most part, very concrete and prosaic in what it was addressing.

Theory-based evaluations that draw upon the realist evaluation approach take it as axiomatic that context is a key mediator between desired objectives and actual outcomes. Context contains numerous dimensions and is not easily demarcated, but in the case of our evaluation, its impact is seen in at least 3 levels: social, organizational, and individual. A further crucial dimension of RE is the mechanism, or what might be deemed the underlying causality that explains why certain actions lead to particular outcomes. The overarching model for RE is context-mechanism-outcome (CMO) configurations, that is, the causal but often convoluted relationship between conditions and outcomes. In undertaking RE , therefore, we have sought to identify and gauge which contextual factors have influenced the outcomes, whether these contexts were anticipated in the design of the intervention, and to what extent mechanisms of change imagined at the outset were confirmed in the outcomes.

Ethical approval for the study was obtained from Region MidtJylland’s research board (May 18, 2018). All data extracted from the DIAVIB group were anonymized before being put to use. Likewise, all interview participants were required to sign an informed consent form, guaranteeing their anonymity but allowing researchers unhindered access to the interview transcripts.

## Results

### Overview

In the period observed, the administrator of the site initiated 109 unique communication threads across a wide range of subjects related to diabetes. In 30 of these threads, the message was accompanied by a link to some external source of information. In 14 cases, the administrator initiated a thread to conduct a poll among the members of DIAVIB. The 109 threads received a total of 780 likes from members of the group, and there were 232 follow-up comments. Many members of the group commented on multiple occasions and the 232 comments were authored by 76 individual members of the group.

The topics attracting the most likes and comments were related to both general diabetes information and information pertaining specifically to the clinic. New developments in diabetes technology were by far the most popular posts in relation to general diabetes information, judged by number of likes and comments. For example, a post about the implantable glucose sensor Eversense XL (Senseonics Holdings) received 54 likes and was commented on 25 times. Of the information specifically pertaining to the clinic, it was personal information about staff members joining or leaving the team that was the most popular on the metric of likes and comments. For example, a thread about a nurse who was leaving the clinic to take retirement received 44 likes and 18 comments.

At the outset of the project, we identified 5 objectives that represented the underlying program theory of DIAVIB. We have subsequently discarded one of these objectives, relating to peer support, on the basis that the setup of DIAVIB was not actually suited to facilitate peer support and the data we acquired from participants reflected this fact. As such, it was deemed to be something that could not be reasonably evaluated. From the 4 remaining objectives, we posited 4 different CMO configurations.

### CMO 1: DIAVIB as a Source of Knowledge

A nonexhaustive summary of this process is exemplified in table form, as seen in Table 2 for the CMO configuration, DIAVIB as a source of knowledge.

**Table 2:** Context-mechanism-outcome configuration 1: Diabetes Viborg as source of knowledge about diabetes.

CMO^a^	Objective	Context	Mechanism	Outcome
CMO 1: Source of knowledge	DIAVIB^b^ should provide people with a reliable source of knowledge about diabetes.	Individual: People with diabetes and their relatives. Social: Information landscape of diabetes (internet, social media, popular media, etc).	People feel overwhelmed by amount of available information about diabetes and have doubts about its veracity. People trust the knowledge and integrity of their HCPs^c^ and will attach value and validity to information provided by their clinic on Facebook.	DIAVIB is used as a primary information source about diabetes by its users. Anxiety/distress related to diabetes information is reduced.

^a^CMO: context-mechanism-outcome.

^b^DIAVIB: Diabetes Viborg.

^c^HCPs: health care professionals.

In our interview data, participants did express concerns relating to the volume of information about diabetes, both in general and on the internet, and the challenge of determining its veracity:

I’ve been on the internet and looked at different things, but I think people say a lot of different things there. Some say something, and others say something else. That can make things all a bit more confusing.

Woman with type 2 diabetes, aged 64 years

The extent to which this was viewed as a problem varied, but there was a clear distinction in the degree to which people with diabetes viewed it as a problem and the degree to which health care professionals saw it as such. Rightly or wrongly, people with diabetes did not experience it as essentially problematic because they felt able, in one way or another, to find a way to normalize things for themselves:

Once you’ve had it for a while you get more and more information, so you just learn. I don’t think there is too much.

Man with type 2 diabetes, aged 66 years

I’ve learnt to filter it out. I’ve grown up with diabetes, so I know what I need to relate to.

Woman with type 1 diabetes, aged 31 years

If there is anything you are in doubt about then you can always look it up. You can look up everything these days.

Woman with type 1 diabetes, aged 40 years

In contrast, every HCP interviewed expressed concern about people being exposed to inaccurate information and the consequences this might have.

However, while interview participants did not indicate a sense of being overwhelmed by information, there was recognition that information provided through DIAVIB did carry extra credibility compared with other more random sources. In fact, for some participants, their contact with the clinic was perceived to provide them with all the information that they needed about diabetes:

I get [information about diabetes] from here [the clinic]. It's not something I read about. If you start to read about it, you will immediately get 10 more symptoms and I don’t want that. I trust what they do here, and I do what they say and I’m fine with that.

Man with type 2 diabetes, aged 67 years

### CMO 2: Forum for Patient-Provider Interaction

The next CMO configuration we identified was DIAVIB as a forum for patient-provider interaction, exemplified in Table 3.

**Table 3:** Context-mechanism-outcome configuration 2: forum for patient-provider interaction.

CMO^a^	Objective	Context	Mechanism	Outcome
CMO 2: Forum for interaction	DIAVIB^b^ should be a forum in which people with diabetes and HCPs^c^ can interact with one another.	Individual: People with diabetes and their relatives; HCPs working with people with diabetes. Social: Juridical system, health care ethics, professional cultures, etc. Organizational: Work organization, task accreditation.	People with diabetes are interested in communicating with their HCPs in online forums because they have needs and concerns that are not addressed in the conventional point of contact with the health care system. HCPs can provide cost-effective support to people with diabetes via online interaction, which will also provide insight into the prevailing concerns among people with diabetes.	People with diabetes and HCPs use DIAVIB to interact with one another. DIAVIB can be used to communicate with patients in a way that supports mutually beneficial and progressive patient-provider interaction.

^a^CMO: context-mechanism-outcome.

^b^DIAVIB: Diabetes Viborg.

^c^HCPs: health care professionals.

The possibility of two-way communication between HCPs and people with diabetes was, in principle, something that could be facilitated by DIAVIB. However, this possibility was limited by the fact that it was only the administrator of the group (the consultant) who could initiate posts. So, while it was possible for members to comment on posts, they were not able to determine the topics under discussion. For some, this represented a limitation that lessened the appeal of engaging with the group:

Yes, I think it would be a good thing. I know that there are other Facebook groups with people who share experiences, so ... for me it’s not likely I’d join the group if they only share information because I think that I can do this myself, also with respect to being critical of the sources. So, if there are no elements besides that in the Facebook group, then I don’t think it’s so interesting for me.

Woman with type 1 diabetes, aged 20 years

Others voiced a wish for more communication, expressing dissatisfaction with the way in which dialogue had been handled within DIAVIB:

I think it could be better in the way that, if there are questions in there, then they should make sure to answer them. They should be a bit more active. Sometimes there are long gaps before anything gets posted.

Woman with type 1 diabetes, aged 59 years

In general, however, there was uncertainty about opening up DIAVIB to more direct two-way communication, expressed as a concern about the type and quality of exchanges that would ensue:

I think it’s a professional tool. I think it’s important that the things that get written are based on professional knowledge. The things posted in here should come from doctors or nurses, so there isn’t any misunderstanding about what is and isn’t true.

Woman with type 1 diabetes, aged 50 years

It was also seen as open to question what kind of communication someone would want to have:

Is this really the right forum, if I’ve got a need to get in touch with my Doctor? Then it would be more about me and not something that I would want to share in an open group.

Woman, with type 1 diabetes, aged 34 years

An underlying factor in the general ambivalence toward the use of DIAVIB as a forum for direct interaction with HCPs could also be inferred from the fact that participants did not express frustration with the degree of contact that they had with HCPs. The interview data presented an overwhelmingly positive impression of a clinic and clinic personnel that were attentive and accessible.

### CMO 3: HCP Engagement With Health Communication

DIAVIB was conceived to inspire HCP engagement with health communication, and the CMO configuration derived from this objective is exemplified in Table 4.

**Table 4:** Context-mechanism-outcome configuration 3: Diabetes Viborg supports health care professional engagement with health communication.

CMO^a^	Objective	Context	Mechanism	Outcome
CMO 3: HCP^b^ engagement with health communication	DIAVIB^c^ should foster an interest in innovative health care communication among HCPs	Individual: HCPs working with people with diabetes. Social: Informed patients, patient-centered care, etc. Organizational: Resources and time dedicated to task.	HCPs are challenged by the expansion of publicly available knowledge and by time limitations in their encounters with people with diabetes.	The opportunities for direct communication offered by DIAVIB will motivate HCPs to engage more in the dissemination of valid and relevant knowledge, which addresses the everyday needs of patients with diabetes.

^a^CMO: Context-mechanism-outcome.

^b^HCP: health care professional.

^c^DIAVIB: Diabetes Viborg.

All the HCPs interviewed acknowledged that the advent of the internet had made some impact on their interactions with people with diabetes. This was viewed as something with both positive and negative consequences. It was, however, primarily the negative consequences that were emphasized by HCPs, who felt that inaccurate information could lead to false expectations and even dangerous actions among people with diabetes. While the notion that there is a need for innovative approaches to health communication is supported in these observations, not all HCPs agreed that posting on Facebook was viable. One concern expressed related to the complexity of the information being conveyed and the challenge of supplying information at a general level, thereby omitting the more personal judgements involved when conveying information to people with diabetes:

I mean, when you’re talking about diet, there’s all sorts of information that you can write about which the patient will see. And I just think, the things we write should be quite specific when you know that a lot of patients are going to read it and you don’t know how they’re going to react to it. That’s something we talk about a lot—what we should and shouldn’t say—where you need to make a judgement based on the individual and that’s just easier when you’re in an individual consultation with the patient.

Dietician, aged 44 years

More prosaically, reservations were voiced in relation to the time needed to maintain the group. Even though the clinic’s personnel were sympathetic to the initiative, it also evoked more negative emotions:

So, it’s a bit like there is a mild pressure to contribute, and that’s fair enough, but it’s like, argh, when is there going to be time for that, to actually sit down and provide something worthwhile… We could do more, but I don’t know when or how it should be.

Dietician, aged 52 years

Although there had been discussions within the clinic about making DIAVIB a collective responsibility, because it was not something that was integrated into the clinic’s everyday practice, it emerged as an exclusively individually driven initiative. DIAVIB was only officially supported to the extent that it existed nominally under the auspices of the regional hospital and clinic, but the time used to set up and maintain the site was not financially reimbursed.

Aside from the issue of time and reimbursement, personnel at the clinic also felt that Facebook imposed limitation in terms of what could be communicated. An important aspect of this related to privacy, both that of the HCP and users of the clinic:

My first thought was, that’s an innovative and visionary initiative. My second thought was, I don’t want to be personally part of that…And I mean, it’s clear that we can’t have personal information. If there’s anything that’s even remotely identifiable they have to use their digital post box, so it just doesn’t work on Facebook. So, I don’t really know what I could help them with, apart from really general information, like insulin can’t cope with 30°C heat.

Nurse, aged 42 years

The personnel interviewed in our evaluation were aware of the innovative and unrealized potential of Facebook to communicate, such as the consultant who imagined it might serve to capture the hardly reached:

I thought it might be a good way to reach those people who don’t come [to the clinic], but who are always on their Facebook pages, you know.

Doctor, aged 38 years

Despite this, interviews with the clinic’s personnel ultimately left an overriding sense of DIAVIB falling short in activating the potential to reach hardly reached patients, a feeling captured by the same consultant reflecting on his own idea:

So that could be a way to get some more people in. But, it’s not really integrated into my way of working. It’s not like I sit here and say, you should do this and this and you can see it on our Facebook group. I’m not there yet… I don’t know, maybe it’s because I don’t really use Facebook much myself.

Doctor, aged 38 years

### CMO 4: Improved Empowerment and Outcomes

The final CMO configuration we identified anticipated that DIAVIB would help patients to achieve improved empowerment and outcomes, as exemplified in Table 5.

**Table 5:** Context-mechanism-outcome configuration 4: Diabetes Viborg improves empowerment and clinical outcomes.

CMO^a^	Objective	Context	Mechanism	Outcome
CMO 4: Improves empowerment and outcomes	DIAVIB^b^ should enable people with diabetes to achieve an improved illness understanding.	Individual: People with diabetes and their relatives. Social: Knowledge sharing.	People’s diabetes management practices are related to their level of knowledge about diabetes. Enhanced knowledge will enable improved diabetes management.	People with diabetes in the clinic will become better at diabetes management and thereby improve their diabetes-related outcomes (eg, HbA_1c_^c^)

^a^CMO: context-mechanism-outcomes.

^b^DIAVIB: Diabetes Viborg.

^c^HbA_1c_: glycosylated hemoglobin, type A_1c_.

This CMO configuration was ultimately the most abstract to evaluate, since there were no means by which to measure participants’ level of engagement with DIAVIB and equate it to changes in clinical outcomes. It was, however, possible to investigate the premise for the proposed mechanism of change and find support for the notion in principle:

You should try to know more about your illness. Knowing more about it, you’re better able to control it.

Man with type 2 diabetes, aged 70 years

At the same time, we also identified individual strategies relating to diabetes knowledge that pushed in the opposite direction. Information overload in relation to diabetes does not only come from what one can hear and read about it. Dealing with diabetes on a day-to-day basis can also be experienced as a type of information overload. In view of the potentially endless information that is available, it is also important that people can delimit what they do and do not need to know:

Interviewee: *I know that I could read a whole lot more about diabetes, but there are just so many other things that I would rather do.* [Woman with type 1 diabetes, aged 40]
Interviewer: *Yeah, life is about more than diabetes?*
Interviewee: *Yeah, where I just think that if there is something that I need to know, well, then I’ll take an interest in it. And if I don’t need to know about it, then I don’t see any reason to take an interest in it.*

## Discussion

### Summary of Findings

Our evaluation of DIAVIB indicates that while acceptance of the initiative was apparent in the numbers who joined the group and the overall positive attitude expressed during the interviews, levels of direct engagement were much lower. This possibly reflects more fundamental challenges in health care communication, where there is generally a lack of clear guidelines for how best to generate content and strategies for communication and engagement with people living with chronic health conditions such as type 1 and type 2 diabetes [29]. At the same time, there are more specific challenges related to designing Facebook groups and pages that are acceptable to all relevant stakeholders [29], not least in achieving congruency about what the purpose is.

Although many HCPs express concerns about the veracity of online information in general, there is no overwhelming evidence that clinically inaccurate information is flooding online diabetes forums [34]. The interview data we obtained did not support a view of people feeling overwhelmed by information and not knowing what to believe. In a recent published commentary, the authors proposed that sifting through the plethora of diabetes-related online information and determining what is and is not meaningful is more of an art form than a scientific process [35]. Although the authors also suggest that greater engagement by HCPs in guiding people with diabetes through this minefield might improve the situation, it is likely that some level of individual interpretation will remain. For better or worse, “patienthood” is becoming a more and more skilled practice [36]. Our informants were happy to use DIAVIB as a source of knowledge about diabetes, and their familiarity with the real-world context in which it was being produced inclined them to ascribe high levels of credibility to the information. At the same time, however, this was something that they generally experienced as nice to have and not as something that they needed to have.

The nature of the communication on DIAVIB was also influenced by the setup of the group (ie, a closed Facebook group associated with a physical diabetes outpatient clinic in a regional hospital, in which only the administrator can initiate topics for discussion and which is primarily being maintained by one individual, for the most part as a hobby rather than something being officially recognized and rewarded). These architectural affordances of the group inevitably impact the type and level of interaction and the respective roles of people with diabetes and HCPs [37]. Rather than transforming modes of interaction between patients with diabetes and HCPs, the architectural affordances of DIAVIB tend to recapitulate them [38]. The empowering potential of social media is, in this sense, somewhat constrained, and a more open architecture within the group may have offered different types and patterns of communication.

Online interaction between patients and providers has previously been shown to be problematic, with a discrepancy between the concerns being voiced by patients and the nature of replies being provided by HCPs, particularly in regard to the use of inclusive and supportive language [39]. Interviews with HCPs indicated that there were concerns about finding the right tone in potential online communication with people with diabetes, especially in the absence of social cues that they would use to tailor their communication and advice in face-to-face encounters. This would support the idea that the advantages afforded by social media may be best realized in cases where there is a preexisting good relationship between those who are interacting [40]. In our case, however, by far the greatest barrier from the HCP perspective was the fact that there was no official recognition of the initiative, and in the absence of guidelines and earmarked resources, DIAVIB was, from an organizational perspective, an essentially vulnerable initiative primarily supported by the commitment of one individual.

It remains unclear whether participation in online support groups serves to establish collective empowerment or whether the collective identity fostered in such groups only serves to generate individual empowerment [41]. Different media can foster different types of empowerment, and forums such as DIAVIB, which are promoted under the auspices of health care organizations, are probably more conducive to the promotion of individual empowerment. Ultimately, our data did not provide any strong indication of DIAVIB members obtaining a sense of empowerment or, for that matter, seeking to obtain a sense of empowerment as such. Nonetheless, by providing a source of reliable information that can contribute to enhancing people’s illness understanding, individual empowerment, in the sense of being able to make more informed decisions regarding care and treatment, may be something that individuals are able to obtain from social media–mediated interaction with their HCPs. This is, however, a question which requires more systematic investigation, although identifying strong evidence for a direct link between participation in groups such as DIAVIB and improved clinical outcomes is likely to remain elusive.

At the current time, there remain concerns about whether the advance of social media and its increasing pervasiveness in all aspects of life may also engender a situation in which certain groups of people are actually disempowered. This applies, for example, in the case of engaging older people with diabetes via social media, where more support is often needed to overcome the barriers they experience [42]. Low health literacy is also negatively associated with ability to accurately assess the quality of online health information [43], and although this is also an issue more generally, in health care there may be specific contours of eHealth literacy [44] that need to be attended to in the case of social media–mediated interactions between people with diabetes and their HCPs.

DIAVIB was very clearly a complex intervention involving various stakeholders located in diffuse contexts and, as such, it was suited to a theory-driven approach to its evaluation. For reasons highlighted above, we adopted a realist evaluation framework to structure our investigation. RE’s approach is not always easy to follow in relation to mechanisms of change, and it assumes a rationality regarding these mechanisms that is not necessarily in place [45]. There are, moreover, diverging views regarding the nature of “mechanism” and the difference between mechanism and essential context condition [46]. However, acknowledging these challenges, the framework provided by RE has also provided clear benefits. Dealing with an intervention that emerged organically, the RE approach compelled both the program developer and the evaluators to explicate the underlying theoretical framework. This exercise had clear value to the task at hand, subsequently framing the analytical focus, for example, in the iteration of interview guides. At the same time, it also gave cause for more general reflection on the criteria by which initiatives such as DIAVIB need to be assessed and the mechanisms and contexts that are likely to influence the success or failure of such initiatives.

### Conclusion

DIAVIB was an initiative that was inspired by motives rooted in genuine and contemporary concerns about supporting people with diabetes in the best possible way. It sought to exploit the potential for new modes of patient-provider interaction seemingly allowed by social media and, at the same time, aimed to provide support for people with diabetes in a world in which flows of information are not necessarily anchored in conventional understandings of knowledge and truth. However, from the perspective of the objectives it was anticipated to address, the success of the initiative is limited. Part of this rests in the expectations, which were highly ambitious. Social media serves well as a conduit for imagining positive change, but this can be a strength and weakness when attempting to enact change via concrete interventions. This is especially true of initiatives like DIAVIB, which are developed organically rather than systematically.

Having stressed the limited extent to which DIAVIB represents a successful intervention when seen through the lens of a theory-driven evaluation, it should finally be noted that such an evaluation does not necessarily capture more intangible benefits. Whatever its limitations, the fact that more than 600 individuals have actively sought membership in DIAVIB suggests that it has tapped into a seam of interest in the possibilities allowed by social media in the context of health care that, as of yet, are not fully realized. Although our study suggests that there remain numerous and serious obstacles on the path towards the realization of such potential, pioneering initiatives such as DIAVIB and the lessons that can be drawn from them represent important milestones along this seemingly inexorable route.

## Abbreviations

CMO: context-mechanism-outcome
DIAVIB: Diabetes Viborg
DOC: diabetes online community
HCP: health care professional
RE: realist evaluation

## References

[1] Riddle MC, Herman WH (2018). The Cost of Diabetes Care-An Elephant in the Room. Diabetes Care.

[2] Williams R, Karuranga S, Malanda B, Saeedi P, Basit A, Besançon S, Bommer C, Esteghamati A, Ogurtsova K, Zhang P, Colagiuri S (2020). Global and regional estimates and projections of diabetes-related health expenditure: Results from the International Diabetes Federation Diabetes Atlas, 9th edition. Diabetes Res Clin Pract.

[3] Bommer C, Sagalova V, Heesemann E, Manne-Goehler J, Atun R, Bärnighausen Till, Davies J, Vollmer S (2018). Global Economic Burden of Diabetes in Adults: Projections From 2015 to 2030. Diabetes Care.

[4] Moorhead SA, Hazlett DE, Harrison L, Carroll JK, Irwin A, Hoving C (2013). A new dimension of health care: systematic review of the uses, benefits, and limitations of social media for health communication. J Med Internet Res.

[5] Fisher J, Clayton M (2012). Who gives a tweet: assessing patients' interest in the use of social media for health care. Worldviews Evid Based Nurs.

[6] Ventola CL (2014). Social media and health care professionals: benefits, risks, and best practices. P T.

[7] Benetoli A, Chen T, Aslani P (2018). How patients' use of social media impacts their interactions with healthcare professionals. Patient Educ Couns.

[8] Shaw RJ, Johnson CM (2011). Health Information Seeking and Social Media Use on the Internet among People with Diabetes. Online J Public Health Inform.

[9] Koteyko N, Hunt D (2016). Performing health identities on social media: An online observation of Facebook profiles. Discourse, Context & Media.

[10] Ruckenstuhl P, Schippinger M, Liebmann P, Leithner A, Bernhardt G (2016). Like or Dislike? Impact of Facebook on Ewing Sarcoma Treatment. JMIR Cancer.

[11] O'Keeffe DT, Montori VM (2016). What's up #DOC? The role of social media in diabetes management. Diabet Med.

[12] Kind T (2015). Professional guidelines for social media use: a starting point. AMA J Ethics.

[13] Centola D (2013). Social Media and the Science of Health Behavior. Circulation.

[14] Oser TK, Oser SM, Parascando JA, Hessler-Jones D, Sciamanna CN, Sparling K, Nease D, Litchman ML (2020). Social Media in the Diabetes Community: a Novel Way to Assess Psychosocial Needs in People with Diabetes and Their Caregivers. Curr Diab Rep.

[15] Litchman ML, Walker HR, Ng AH, Wawrzynski SE, Oser SM, Greenwood DA, Gee PM, Lackey M, Oser TK (2019). State of the Science: A Scoping Review and Gap Analysis of Diabetes Online Communities. J Diabetes Sci Technol.

[16] Litchman M, Edelman LS, Donaldson GW (2018). Effect of Diabetes Online Community Engagement on Health Indicators: Cross-Sectional Study. JMIR Diabetes.

[17] Tenderich Amy, Tenderich Burghardt, Barton Tanner, Richards Sarah Elizabeth (2019). What Are PWDs (People With Diabetes) Doing Online? A Netnographic Analysis. J Diabetes Sci Technol.

[18] Langstrup H, Rahbek AE (2015). Conceptualizing ‘role’ in patient-engaging e-health: A cross-disciplinary review of the literature. Commun Med.

[19] Devi S (2011). Facebook friend request from a patient?. The Lancet.

[20] Moubarak G, Guiot A, Benhamou Y, Benhamou A, Hariri S (2011). Facebook activity of residents and fellows and its impact on the doctor-patient relationship. J Med Ethics.

[21] Giménez-Pérez Gabriel, Recasens A, Simó Olga, Aguas T, Suárez Ana, Vila M, Castells I (2016). Use of communication technologies by people with type 1 diabetes in the social networking era. A chance for improvement. Prim Care Diabetes.

[22] Lyles Courtney R, López Andrea, Pasick R, Sarkar U (2013). "5 mins of uncomfyness is better than dealing with cancer 4 a lifetime": an exploratory qualitative analysis of cervical and breast cancer screening dialogue on Twitter. J Cancer Educ.

[23] Benetoli A, Chen TF, Schaefer M, Chaar B, Aslani P (2017). Do pharmacists use social media for patient care?. Int J Clin Pharm.

[24] Andersen Kn, Medaglia R, Henriksen Hz (2012). Social media in public health care: Impact domain propositions. Government Information Quarterly.

[25] Househ M, Borycki E, Kushniruk A (2014). Empowering patients through social media: the benefits and challenges. Health Informatics J.

[26] Zhang Y, He D, Sang Y (2013). Facebook as a platform for health information and communication: a case study of a diabetes group. J Med Syst.

[27] Petrovski G, Zivkovic M (2017). Impact of Facebook on Glucose Control in Type 1 Diabetes: A Three-Year Cohort Study. JMIR Diabetes.

[28] Duke DC, Barry S, Wagner DV, Speight J, Choudhary P, Harris MA (2018). Distal technologies and type 1 diabetes management. The Lancet Diabetes & Endocrinology.

[29] Partridge SR, Gallagher P, Freeman B, Gallagher R (2018). Facebook Groups for the Management of Chronic Diseases. J Med Internet Res.

[30] Marchal B, van Belle S, van Olmen J, Hoerée T, Kegels G (2012). Is realist evaluation keeping its promise? A review of published empirical studies in the field of health systems research. Evaluation.

[31] Pawson R, Tilley N (1997). Realistic Evaluation.

[32] Ranmuthugala G, Cunningham FC, Plumb JJ, Long J, Georgiou A, Westbrook JI, Braithwaite J (2011). A realist evaluation of the role of communities of practice in changing healthcare practice. Implement Sci.

[33] Blamey A, Mackenzie M (2016). Theories of Change and Realistic Evaluation: Peas in a Pod or Apples and Oranges?. Evaluation.

[34] Greene JA, Choudhry NK, Kilabuk E, Shrank WH (2011). Online social networking by patients with diabetes: a qualitative evaluation of communication with Facebook. J Gen Intern Med.

[35] Reidy C, Klonoff DC, Barnard-Kelly KD (2019). Supporting Good Intentions With Good Evidence: How to Increase the Benefits of Diabetes Social Media. J Diabetes Sci Technol.

[36] Kingod N, Cleal B, Wahlberg A, Husted GR (2017). Online Peer-to-Peer Communities in the Daily Lives of People With Chronic Illness: A Qualitative Systematic Review. Qual Health Res.

[37] Årsand Eirik, Bradway M, Gabarron E (2019). What Are Diabetes Patients Versus Health Care Personnel Discussing on Social Media?. J Diabetes Sci Technol.

[38] Hunt D, Koteyko N (2015). ‘What was your blood sugar reading this morning?’ Representing diabetes self-management on Facebook. Discourse & Society.

[39] Alpert JM, Dyer KE, Lafata JE (2017). Patient-centered communication in digital medical encounters. Patient Educ Couns.

[40] Huxley CJ, Atherton H, Watkins JA, Griffiths F (2015). Digital communication between clinician and patient and the impact on marginalised groups: a realist review in general practice. Br J Gen Pract.

[41] Wentzer HS, Bygholm A (2013). Narratives of empowerment and compliance: studies of communication in online patient support groups. Int J Med Inform.

[42] Silver MP (2015). Patient perspectives on online health information and communication with doctors: a qualitative study of patients 50 years old and over. J Med Internet Res.

[43] Diviani N, van den Putte B, Giani S, van Weert JC (2015). Low health literacy and evaluation of online health information: a systematic review of the literature. J Med Internet Res.

[44] Kayser L, Karnoe A, Furstrand D, Batterham R, Christensen KB, Elsworth G, Osborne RH (2018). A Multidimensional Tool Based on the eHealth Literacy Framework: Development and Initial Validity Testing of the eHealth Literacy Questionnaire (eHLQ). J Med Internet Res.

[45] Greenhalgh T, Humphrey C, Hughes J, Macfarlane F, Butler C, Pawson R (2009). How do you modernize a health service? A realist evaluation of whole-scale transformation in London. Milbank Q.

[46] Dalkin SM, Greenhalgh J, Jones D, Cunningham B, Lhussier M (2015). What's in a mechanism? Development of a key concept in realist evaluation. Implement Sci.

